# The association of alanine aminotransferase and diabetic microvascular complications: A Mendelian randomization study

**DOI:** 10.3389/fendo.2023.1104963

**Published:** 2023-01-19

**Authors:** Yaru Bi, Yanjing Liu, Heyuan Wang, Suyan Tian, Chenglin Sun

**Affiliations:** ^1^ Department of Endocrinology and Metabolism, First Hospital of Jilin University, Changchun, China; ^2^ Department of Medicine, Lvyuan People’s Hospital, Changchun, China; ^3^ Division of Clinical Research, First Hospital of Jilin University, Changchun, China; ^4^ Department of Clinical Nutrition, First Hospital of Jilin University, Changchun, China

**Keywords:** alanine aminotransferase, diabetic nephropathy, diabetic retinopathy, diabetic microvascular complication, Mendelian randomization

## Abstract

**Aims:**

Alanine aminotransferase (ALT) is positively related to diabetes risk in observational studies, whereas Mendelian randomization supports a linear causal association. In contrast, the relationship between ALT and diabetic nephropathy, and diabetic retinopathy is counter-intuitive in observational studies. Furthermore, no MR study has examined their causal association. The study aimed to investigate whether genetically determined ALT has a causal effect on diabetic nephropathy and diabetic retinopathy.

**Methods:**

Genetic instruments associated with ALT (*P* < 5×10^-8^) were obtained from a recent genome-wide association study (GWAS) that included 437,267 individuals of European ancestry. Summary data of diabetic microvascular complications were derived from the FinnGen study (3,283 cases and 181,704 controls for diabetic nephropathy, and 14,584 cases and 176,010 controls for diabetic retinopathy, both were of European ancestry). Effect estimation and pleiotropy testing were performed using inverse variance weighted (IVW), MR-Egger regression, weighted median, and mode-based estimator methods. We additionally performed sensitivity analysis excluding proxy single nucleotide polymorphisms (SNPs) or lowering the GWAS significance threshold (*P* < 5×10^-7^) to test the robustness of the results.

**Results:**

Based on IVW, a 2-fold increase in genetically determined ALT level was positively associated with diabetic nephropathy (odd ratio, [95% confidence interval], 1.73 [1.26-2.37], *P* = 0.001) and diabetic retinopathy (1.29 [1.08-1.54], *P* = 0.005), but a null causal association in three pleiotropy robust methods, namely, MR-Egger, weighted median and mode-based estimator. We obtained similar results in the sensitivity analysis of excluding proxy SNPs or lowering the GWAS significance threshold.

**Conclusions:**

With caution, we concluded that ALT plays no linear causal role in developing both diabetic nephropathy and diabetic retinopathy. Further investigations are required to test the hypothesis of a non-linear causal association.

## Introduction

Diabetes mellitus (DM) has become a severe chronic disease worldwide, with an increasing prevalence. It is estimated that 783.2 million adults would have DM by 2045 (1). Thus, the number of DM with microvascular complications, such as diabetic nephropathy and diabetic retinopathy, is also dramatically increasing. Diabetic nephropathy is characterized by microalbuminuria, followed by a decrease in the glomerular filtration rate. Approximately one-third of patients with DM eventually develop diabetic nephropathy (2). It has become the primary cause of end-stage renal disease worldwide and is also a risk factor for cardiovascular disease. The global prevalence of diabetic retinopathy is 34.6%, making it a common complication of diabetes (3). Apart from causing blindness in the elderly (4), diabetic retinopathy also poses a serious risk of systemic vascular complications (5). Therefore, it is critical to identify the risk factors of diabetic nephropathy and diabetic retinopathy, aiming at early prevention and intervention of these complications.

Alanine aminotransferase (ALT) within a certain range is found to be linearly positively correlated with the risk of diabetes in observational studies (6–8). However, when diabetes progresses to the microvascular complications stage, few studies examine its correlation with ALT. ALT is one of the liver enzymes that is predominately located in the hepatocellular cytosol. It is released into the blood due to all-cause of hepatocellular damage, which is considered a specific marker of liver injury. In addition, ALT also reflects hepatocyte lipid accumulation, such as nonalcoholic fatty liver disease (NAFLD) (9). According to one study, NAFLD increased the risk of diabetic retinopathy and diabetic nephropathy (10). ALT levels were positively related to NAFLD. Hence, it is speculated that ALT might increase the risk of diabetic microvascular complications. However, existing epidemiology studies had counter-intuitive results. For example, an Iranian case-control study found that ALT level in the diabetic retinopathy stage was lower compared to DM without diabetic retinopathy (11), and another study indicated that elevated ALT was associated with the remission of diabetic nephropathy (12).

Mendelian randomization (MR) is a method used for causal inference. Specifically, the genetic variants (e.g., single nucleotide polymorphisms, SNPs) associated with exposure are considered to be instrumental variables (IVs) and used to explore the causal association between the exposure (e.g., ALT) and outcome of interest (e.g., diabetic nephropathy or diabetic retinopathy). The genetic variants are randomly assorted at conception, similar to random allocation in a randomized controlled trial. Thus, MR is independent of confounding factors. Earlier MR studies supported the causal association between ALT and DM (13, 14), but no MR study has focused on the causal association between ALT and specific diabetic complications, such as diabetic nephropathy and diabetic retinopathy.

In the current study, we aimed to investigate causal relationships between ALT and specific diabetic microvascular complications, such as diabetic retinopathy and diabetic nephropathy, by conducting two-sample MR studies using the recent genome-wide association study (GWAS) summary data. Such analysis may help to understand the role of ALT in the development and progression of diabetic microvascular complications.

## Materials and methods

Ethics approval was not required for this study. The paper was prepared with reference to the STrengthening the Reporting of Observational studies in Epidemiology-MR (STROBE-MR) checklist for the study (**Table S1**; 15).

### Study design

**Figure 1:**
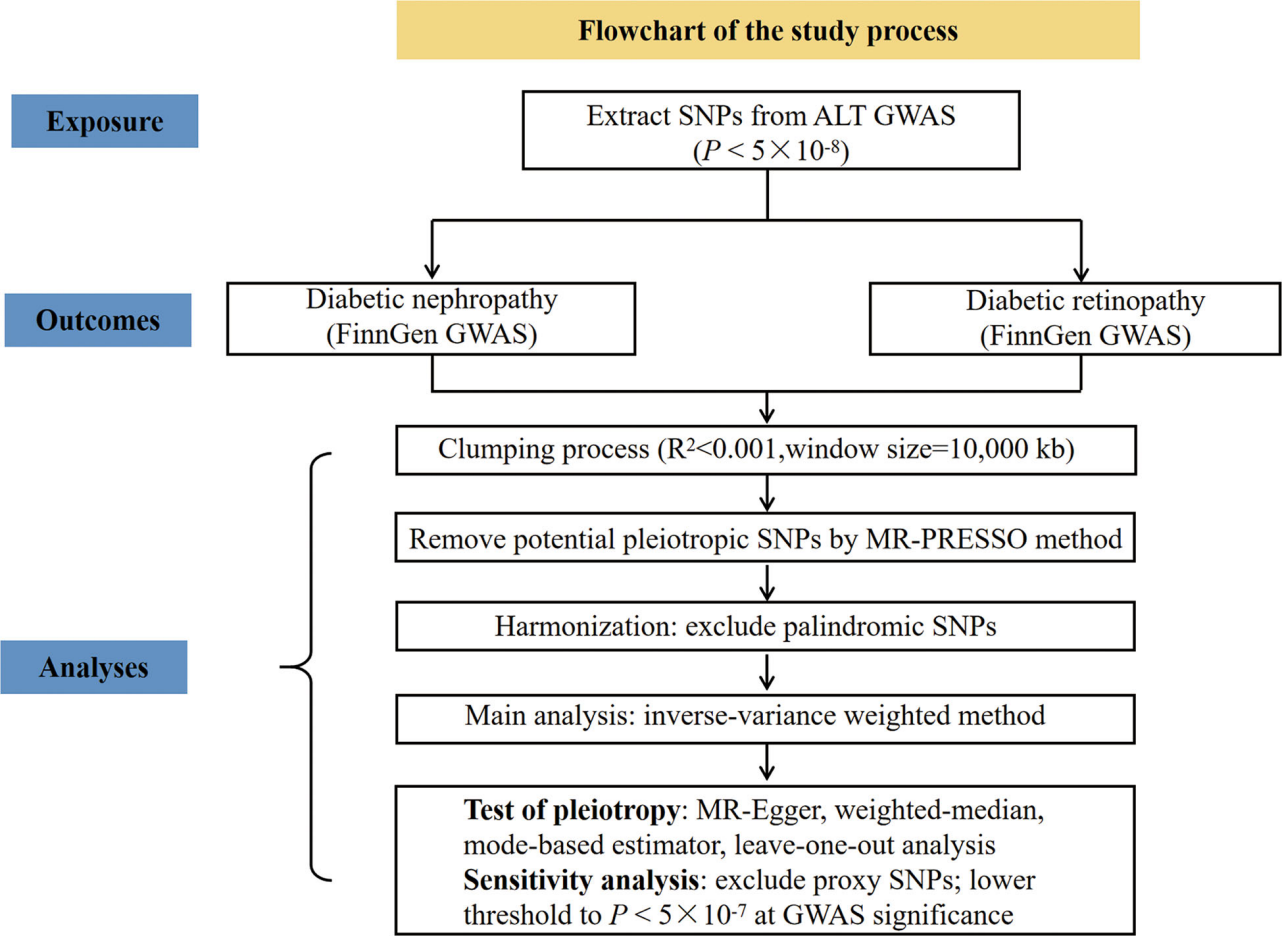
Flowchart of the study process.

To explore the causal role of ALT in diabetic microvascular complications, we conducted two-sample MR analyses with reference to ALT and diabetic nephropathy, ALT and diabetic retinopathy, respectively. The flowchart to illustrate the study process is shown in **Figure 1**.

### Genetic variants associated with ALT

**Figure 2:**
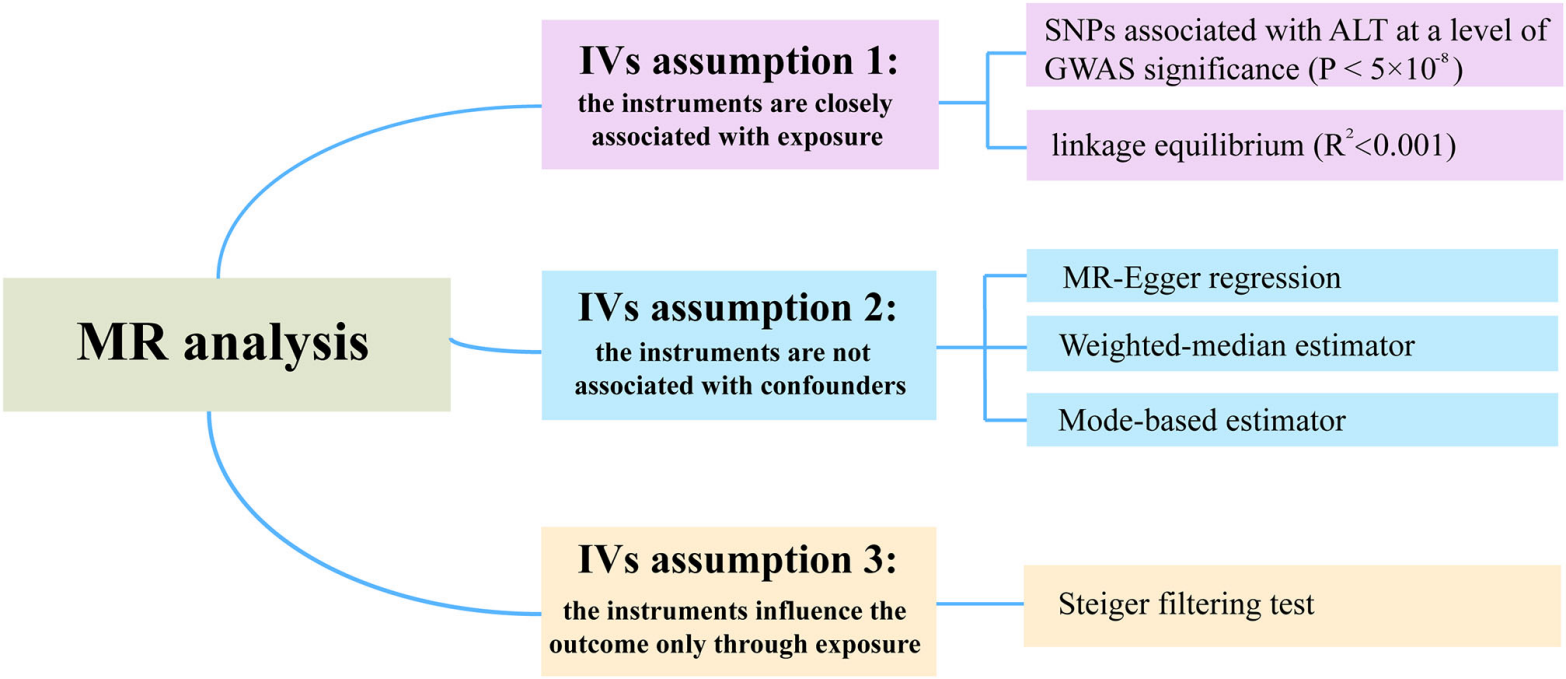
Strategies to satisfy the three assumptions of IVs in MR analysis.

The summary statistics for the association between SNPs and ALT were extracted from a recent GWAS by Pazoki Raha et al, which included 437,267 individuals of European ancestry (16). To justify assumption 1, which requires that the genetic variants are closely associated with the exposure (ALT), we selected SNPs associated with ALT at genome-wide significance (*P* < 5×10^-8^) (**Figure 2**). Moreover, r^2^ < 0.001 (clumping distance=10,000 kb) was used as the threshold to determine that SNPs were in linkage equilibrium. Details on the participants can be found in the related publication (16). F-statistics was calculated by using F = beta^2^/se^2^ (17). The SNPs with F-statistics < 10 were considered as weak IVs and therefore were excluded. Of note, the ALT levels are transformed by log 10 (corresponding to per 10 times of ALT) in the original article, and by multiplying a constant (i.e., log 2/log 10), the scale was modified to log 2, which was consistent with the unit used in the major studies.

### Data sources of diabetic nephropathy and diabetic retinopathy

The summary data for the association between SNPs and diabetic nephropathy or diabetic retinopathy were extracted from the FinnGen consortium released in 2021 (https://r5.finngen.fi/). The GWAS of diabetic nephropathy and diabetic retinopathy comprised 3,283 cases and 181,704 controls, and 14,584 cases and 176,010 controls, respectively, both of European ancestry.

For SNPs associated with ALT that were not found in the diabetic nephropathy or diabetic retinopathy GWAS, we select proxy SNPs that are in linkage disequilibrium (R^2^ > 0.8). The selected SNPs in the exposure-outcome dataset were harmonized to exclude palindromic SNPs.

### Statistical analysis

Wald ratio was first used to evaluate the SNP-specific causal estimation. The conventional inverse variance weighted (IVW) meta-analysis of the Wald ratio for individual SNP, which provided an unbiased causal estimate if the SNPs exhibited no horizontal pleiotropy or balanced horizontal pleiotropy. Fixed and random-effect models of IVW were available. Cochran’s Q test and funnel plots were used to assess heterogeneity. Random-effect models of IVW were applied when the SNPs showed heterogeneity. According to assumption 2 of the IVs, the variants are independent of any confounding factors of the exposure-outcome association. The existence of horizontal pleiotropy in IVs would invalidate this assumption. To test this assumption, we additionally performed three robust pleiotropy methods, such as MR-Egger (18), weighted median (19), and mode-based estimator (20). MR-Egger regression can be used to assess the presence of horizontal pleiotropy based on the intercept of regression line deviation from zero. The estimate given by the weighted median method is still unbiased when up to 50% of IVs are invalid. The mode-based estimator can return an unbiased estimate of the effect of interest even when most IVs are invalid. In addition, we applied MR-PRESSO method (the replicates were set 1000 times) to detect the outliers (21). Then the identified outliers were removed for a reevaluation. According to assumption 3 of IVs, SNPs only influence the outcome through exposure. The Steiger filtering method was used to test the causal direction between two attributes.

### Sensitivity analysis

The leave-one-out sensitivity analysis was performed to determine if a single SNP has a dramatic effect on the association between ALT and the outcome of interest. In addition, we also carried out another two sensitivity analyses. One sensitivity analysis was to lower threshold to *P* < 5×10^-7^ at GWAS significance leading to the inclusion of a larger number of genetic risk variants. Another sensitivity analysis was to exclude the proxy SNPs to reassess the causal estimate.

### Power calculation

mRnd, an online calculator (https://shiny.cnsgenomics.com/mRnd/), was used to calculate the sample size (22). With sample sizes of 184,987 and 190,594 for diabetic nephropathy and diabetic retinopathy, respectively, the powers to detect an odd ratio (OR) of 1.2 for the development of diabetic nephropathy and diabetic retinopathy per standard deviation increase in ALT level (assuming genetic variants explain around 5% of the variance in ALT) are 0.72 and 1.

### Statistical software

All statistical analyses were performed in R software 4.1.0 (https://www.r-project.org/). MR analyses and Steiger filtering were performed using the R package “TwoSampleMR”. The MR-PRESSO was conducted using the R package “MRPRESSO”.

## Results

### SNPs selected as instrumental variables

A total of 190 and 185 SNPs were ultimately included to assess the causal role of ALT in diabetic nephropathy and diabetic retinopathy, with the mean F-statistics being 91.40 and 90.92, respectively, representing that the IVs used in the MR analysis were not weak IVs. Detailed information on the IVs used in the MR analyses is displayed in **Table S2** (diabetic nephropathy) & **Table S3** (diabetic retinopathy).

### Genetic association with diabetic nephropathy

**Figure 3:**
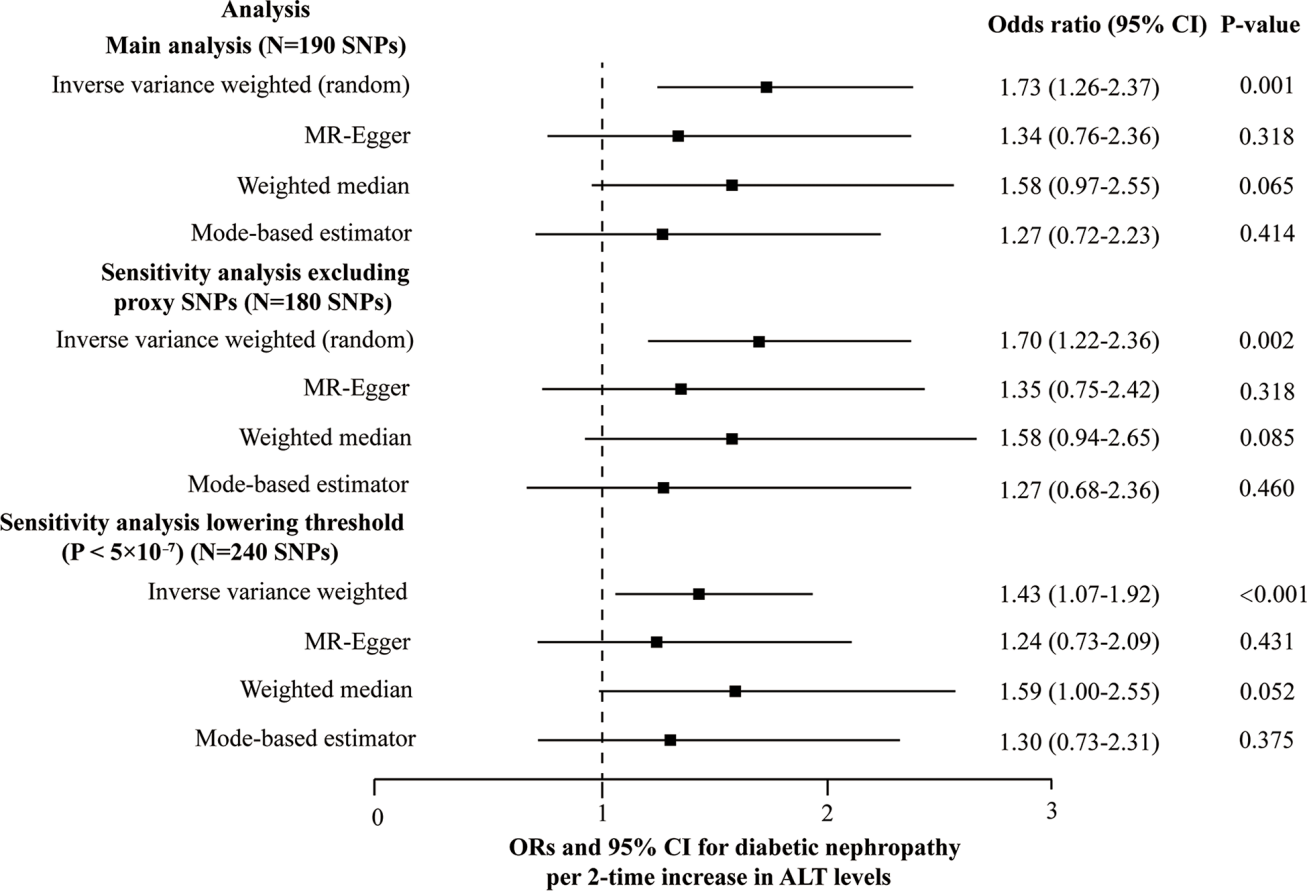
Forest plot of the MR main and sensitivity analyses of ALT and diabetic nephropathy.

Ten out of 190 SNPs are absent in the diabetic nephropathy GWAS and substituted by proxy SNPs with high linkage disequilibrium (r^2^ > 0.8). Due to the heterogeneity across the 190 SNPs based on the heterogeneity test (Cochran’s Q value = 226.6, *P* = 0.032 for MR-IVW, and Q value = 225.3, *P* = 0.033 for MR-Egger) and funnel plot, and IVW (random effects model) was applied. We found that genetically predicted ALT level (2-fold increase) is causally associated with diabetic nephropathy using the IVW with OR 1.73 (95% confidence interval (CI): 1.26-2.37, *P* = 0.001). However, no causal association was observed using MR-Egger (1.34 [0.76-2.36], *P* = 0.318), the weighted median (1.58 [0.97-2.55], *P* = 0.065), and the mode-based estimator (1.27 [0.72-2.23], *P* = 0.414) (**Figure 3**). No indication was detected for unbalanced horizontal pleiotropy (the intercept of the MR-Egger was 0.0047 and the corresponding directionality p-value was 0.288). Based on the leave-one-out analysis, no SNP significantly altered the overall results (Plots of MR estimate, scatter plot, funnel plot, and leave-one-out analysis were displayed in **Figures S1A–D**). SNPs were found to be directed associated with ALT instead of diabetic nephropathy using the Steiger filtering method. Outlier SNPs were not detected using the MR-PRESSO. Besides, considering the marginal statistical significance level for weighted-median method (*P* = 0.065), we further conducted sensitivity analysis through lowering the threshold to *P* < 5×10^-7^ at GWAS significance to include larger genetic risk variants. Another sensitivity analysis was carried out after removing 10 proxy SNPs. Both the sensitivity analyses yielded results similar with those of the main analyses; namely, the IVW method indicated a causal association while the other three showed no causal association between ALT and diabetic nephropathy (**Figure 3** and **Table S4**). In the two sensitivity analyses, no SNPs that significantly influenced the causal association of ALT with diabetic nephropathy were detected using the leave-one-out analysis. The Steiger filtering method verified the direction of the causality should be from ALT to diabetic nephropathy.

### Genetic association with diabetic retinopathy

**Figure 4:**
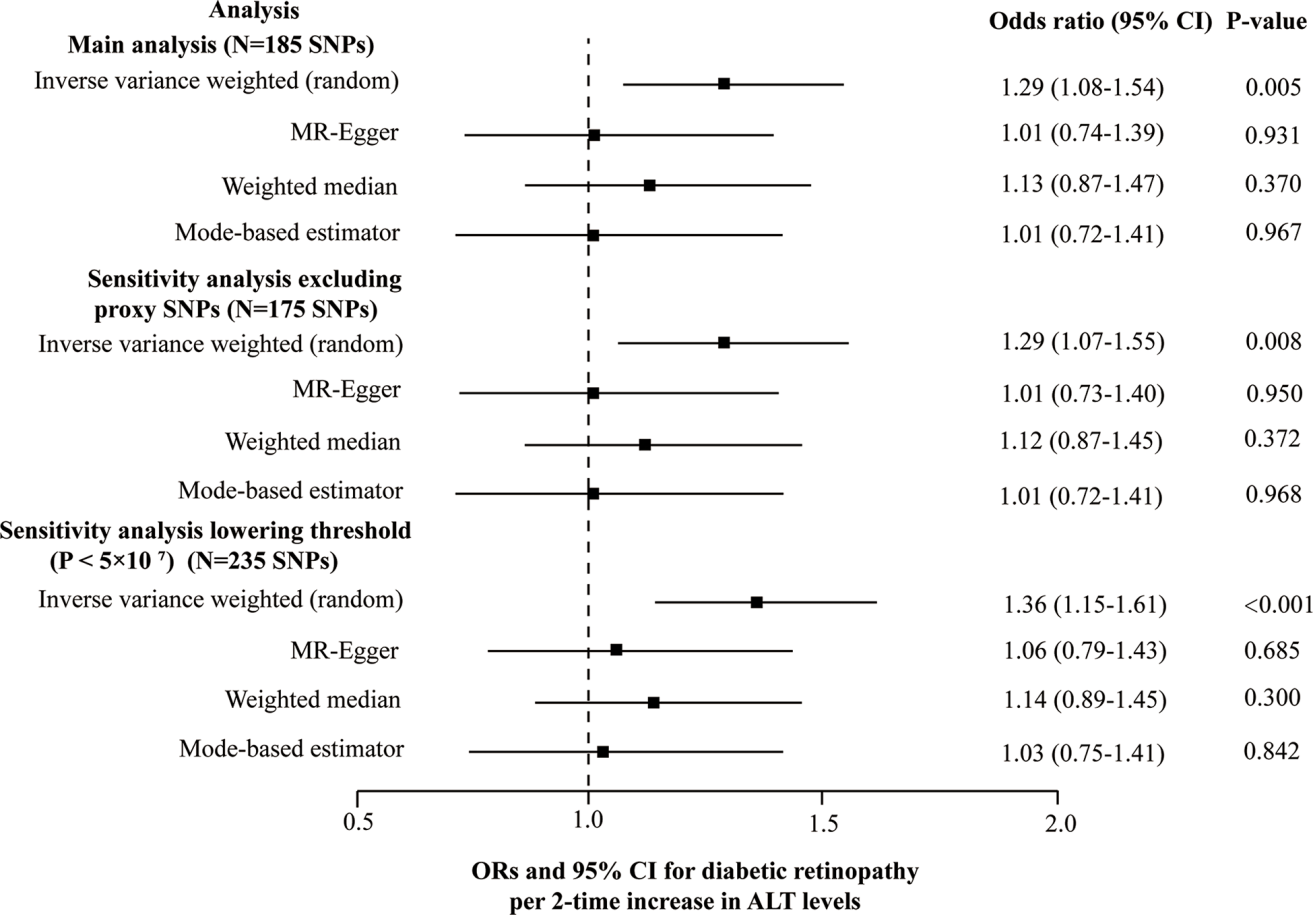
Forest plot of the MR main and sensitivity analyses of ALT and diabetic retinopathy.

5 SNPs (rs3184504, rs429358, rs56094641, rs62523081, rs6695321) were identified as outliers using the MR-PRESSO, and then the outliers were removed to reassess the causal estimate. Finally, our MR analysis included 185 SNPs in total. The heterogeneity test (Cochran’s Q = 254.6, *P* = 3.7E-4 for MR Egger and Q = 259.3, *P* = 2.1E-4 for IVW) and the funnel plot indicated the existence of heterogeneity between 185 SNPs and random-effect model of IVW was fit. No unbalanced horizontal pleiotropy was observed in the SNPs on the diabetic retinopathy (the intercept of the MR-Egger was 0.0045, and the corresponding p-value was 0.068). Genetically determined ALT level (2-fold increase) was causally associated with diabetic retinopathy using the IVW (1.29 [1.08-1.54], *P* = 0.005). However, the other three robust pleiotropy methods yielded different results from the main MR analysis; no causal association was found between ALT and diabetic retinopathy by using MR-Egger (1.01 [0.74-1.39], *P* = 0.931), the weighted median (1.13 [0.87-1.47], *P* = 0.370) and the mode-based estimator (1.01 [0.72-1.41], *P* = 0.967) (**Figure 4**). No SNPs can change the MR results based on a leave-one-out analysis (Plots of MR estimate, scatter plot, funnel plot, and leave-one-out analysis were displayed in **Figures S2A-D**). MR results did not essentially change after lowering the threshold to 5×10^-7^ at GWAS significance or excluding 10 proxy SNPs (**Figure 4** and **Table S5**). At both thresholds (i.e., *P* < 5×10^-8^ at GWAS significance and *P* < 5×10^-7^) results of the Steiger filtering test indicated that direction arrow was from ALT to diabetic retinopathy.

## Discussion

In the present study, a two-sample MR was performed using the summary data of published recent GWAS to analyze the causal association between genetically elevated ALT and diabetic-specific microvascular complications, including diabetic nephropathy and diabetic retinopathy. While a causal association was identified for ALT in correlation with both diabetic nephropathy and diabetic retinopathy using the conventional IVW method, but the other three MR methods (i.e., MR-Egger, the weighted-median, and the mode-based estimator) failed to detect any evident causal interrelation. Considering the different MR results consequent of an unobserved horizontal pleiotropy, we could conservatively conclude that ALT plays no linear causal role in the development of diabetic nephropathy and diabetic retinopathy.

Observational studies detected a decreasing trend of ALT levels in the diabetic nephropathy and diabetic retinopathy stages. For example, one recent observational study enrolled 440 T2DM individuals with at least one microvascular complication (diabetic nephropathy, diabetic retinopathy, and diabetic peripheral neuropathy) and 495 T2DM individuals free of those microvascular complications. The study observations revealed that ALT in the diabetic retinopathy group was lower than in the T2DM patients without any complications (23.9 ± 10.9 IU/L vs 28.7 ± 23.0 IU/L, respectively, *P*<0.05), and there was a decreasing trend of ALT in the diabetic nephropathy group, though not statistically significant (26.7 ± 14.1 IU/L vs 28.7 ± 23.0 IU/L, respectively) (11). Another retrospective study exploring the factors associated with the progression and remission of diabetic nephropathy revealed elevated ALT levels to be associated with the remission of diabetic nephropathy (12). By contrast, ALT levels are found to be increasing in diabetes. A meta-analysis, including 21 studies, indicated that ALT levels (per increase in one unit of logged ALT) could increase the risk of diabetes with a hazard ratio of 1.83 (95% CI: 1.57-2.14) (23).

**Figure 5:**
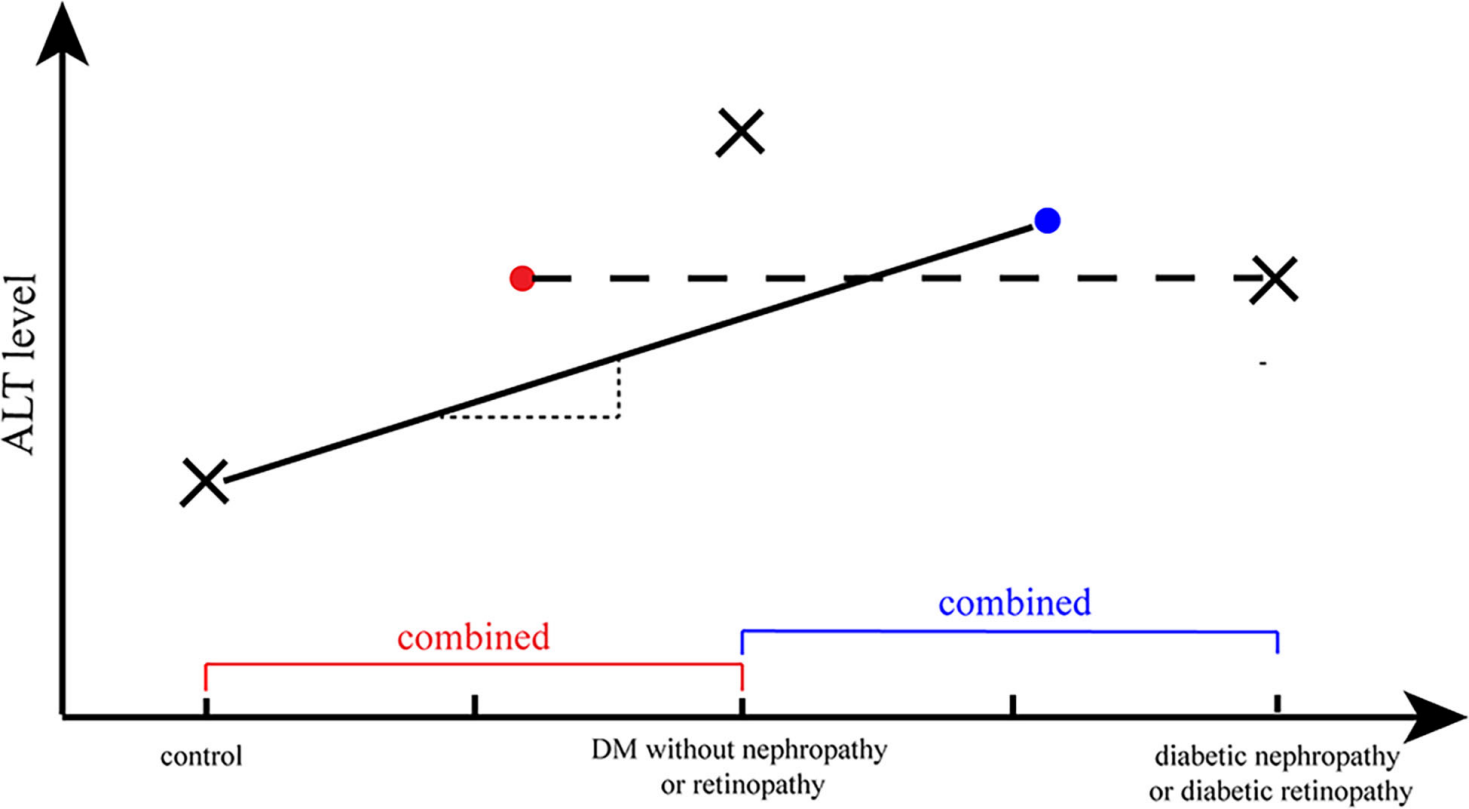
A illustrative diagram to interpret the null causal relation of ALT and diabetic nephropathy/diabetic retinopathy. The cross marks (×) represented the ALT level in different phases of diabetes and the dots (·) represented the average ALT level of a combination group (e.g., the cross mark in the right represented the average level of ALT in individuals with diabetic nephropathy/retinopathy; and the blue dot represented the average level of ALT in DM without diabetic nephropathy/retinopathy group and diabetic nephropathy/retinopathy group). The straight line with a positive slope (representing a positive correlation between the two traits) represented the possible fitted MR analysis of ALT and DM, and the horizontal line (corresponding to no correlation between two attributes) represented the possible fitted MR analysis of ALT and diabetic nephropathy/retinopathy.

We predicted the causal relationship between ALT and diabetic nephropathy/diabetic retinopathy to be non-linear based on the results of previous observational studies. Also, an illustrative diagram was drawn to interpret the presence of a null causal relation between ALT and diabetic nephropathy/diabetic retinopathy in the present focus, where an assumption on linear causal association was made (**Figure 5**). The DM and control groups were compared, with the case group involving DM patients with and without diabetic complications. The mean ALT level for the case group was expected to be between the mean ALT value of DM without complication and that of DM with complications, as represented by the blue dot. As a result, it is possible to fit a straight line with a positive slope (representing a positive correlation between the two traits) (as illustrated by the solid line in **Figure 5**).

On the other hand, ALT levels tended to decline when diabetes gradually progressed to diabetic nephropathy or diabetic retinopathy. The control group consisted of non-DM individuals (normal controls or negative controls) and diabetic individuals without diabetic nephropathy/retinopathy (loosely regarded as positive controls), while comparing the diabetic nephropathy/retinopathy group and the non-diabetic nephropathy/retinopathy group. Consequently, the average ALT levels in the control group should lie in the position of the red dot (**Figure 5**). Thus, a horizontal line should provide an optimal estimate of the relationship between ALT and diabetic retinopathy/diabetic nephropathy, which corresponds to no correlation.

Our study had several advantages. First, to the best of our knowledge, the current MR is one of the pioneer efforts investigating the causal association between ALT levels and diabetic nephropathy/retinopathy. Liu and colleague recently investigated the causal association between NAFLD and diabetic complications, such as diabetic nephropathy and diabetic retinopathy (24), in which the exposure is NAFLD instead of ALT levels. Second, though no linear causal association was found between ALT and diabetic nephropathy/retinopathy, the current study may be considered as a hypothesis generating study, namely, a non-linear causal association between ALT and diabetic microvascular complications was anticipated, which laid a foundation for further non-linear MR analysis. Lastly, compared to previous liver enzyme GWAS study identifying several loci associated with ALT level (25), the latest GWAS, released in 2021, used as the source of ALT in our current MR analysis, identifying more than 200 independent loci closely associated with ALT level, would have a better statistical power.

Our analysis also had several limitations. First, the sample size of diabetic nephropathy GWAS study was inadequately statistically powered (power = 0.72) to detect a small causal effect. However, the FinnGen GWAS is a very recent GWAS that is publicly accessible for studying diabetic nephropathy with the largest sample size. Second, the individuals of the exposure (ALT) are of European ancestry, while the outcomes (diabetic nephropathy and diabetic retinopathy) are from the FinnGen study, which depicts the Finnish population; they are genetically different from conventional European ancestry with respect to LD structure and allele frequency. Third, we are unable to test the hypothesis of the non-linear causal association as we only have GWAS summary statistics for our present MR study. Further non-linear MR analyses are needed to test the research hypothesis of a causal association with stratified MR based on different stages in the development of diabetes using GWAS individual-level data.

In conclusion, we cautiously concluded that there is no causal impact of genetically predicted ALT levels on diabetic nephropathy and diabetic retinopathy. Large prospective studies or future MR analyses based on larger GWAS samples are needed to test the causal association. Notably, based on the results of previous observational and MR studies, and the current MR studies, we hypothesize that a non-linear causal association exists between ALT and diabetic nephropathy/retinopathy. Future investigations, such as stratified MR studies, are highly desirable to test this hypothesis and explore the role of ALT in the development and progression of diabetic microvascular complications.

## Data availability statement

The datasets presented in this study can be found in online repositories. The names of the repository/repositories and accession number(s) can be found in the article/**Supplementary Material**.

## Ethics statement

The studies involving human participants were reviewed and approved by FinnGen Steering Committee. The patients/participants provided their written informed consent to participate in this study.

## Author contributions

CS and ST contributed to conception and design of the study. YB and YL were responsible for writing. YB and HW performed the statistical analysis. All authors contributed to manuscript revision, read, and approved the submitted version.
