# Supplementary material for: The association of alanine aminotransferase and diabetic microvascular complications: A Mendelian randomization study
Source: Front Endocrinol (Lausanne). 2023 Jan 19;14:1104963. doi: 10.3389/fendo.2023.1104963 (PMC9892708; doi:10.3389/fendo.2023.1104963)

Figure S1A. A two-sample MR study of the effect of ALT on diabetic nephropathy.


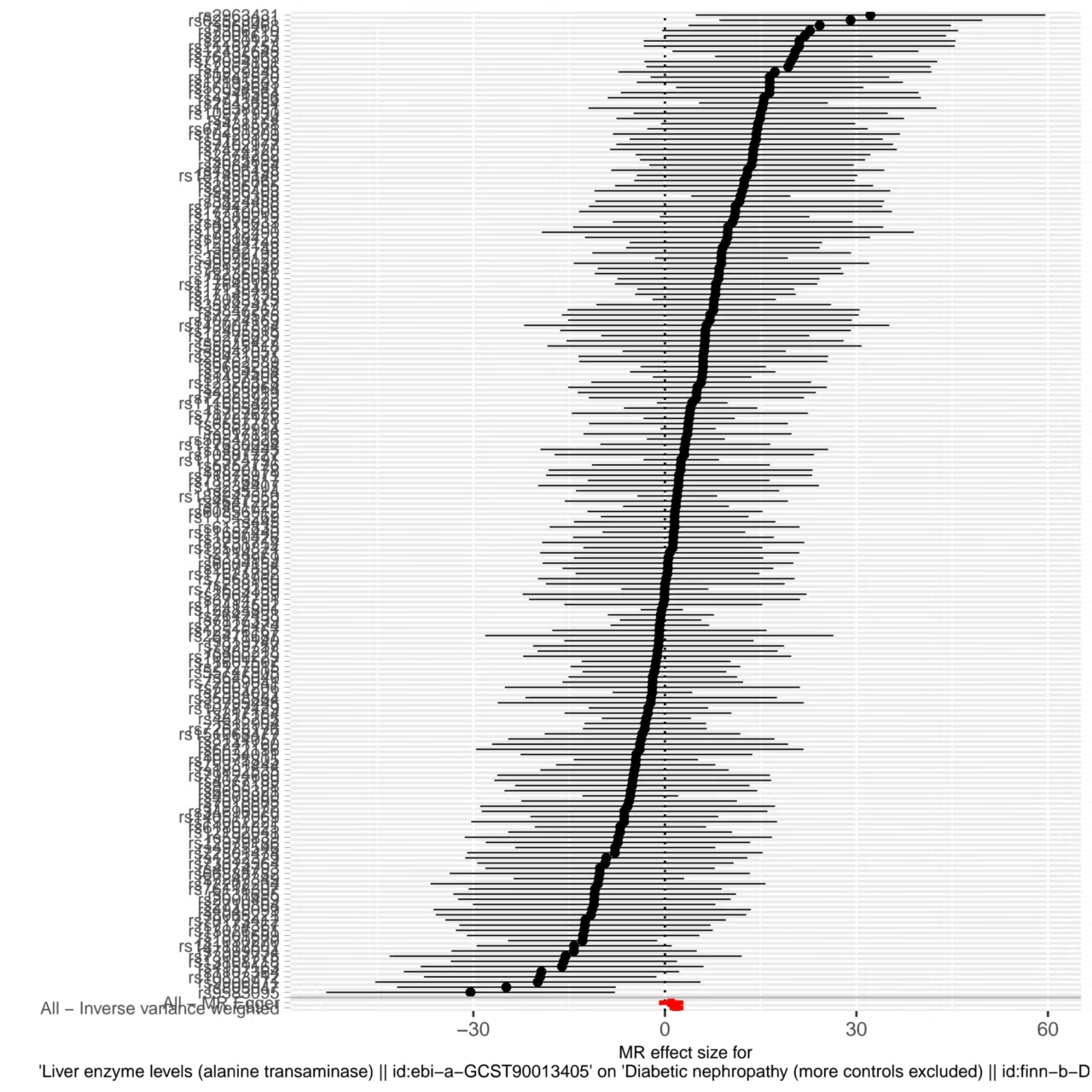


Figure S1B. Scatter plot of the main MR study investigating the effect of ALT on diabetic nephropathy.


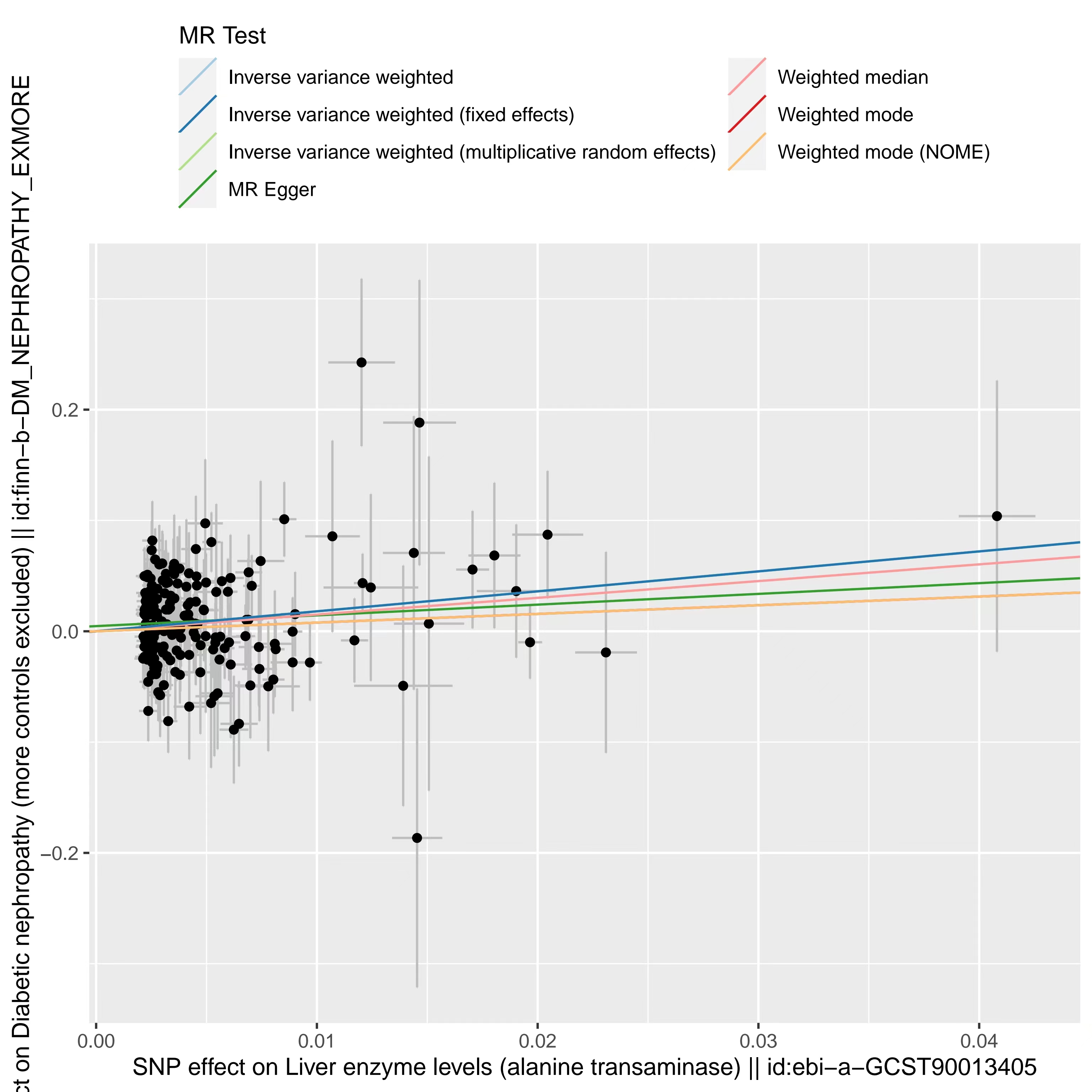


Figure S1C. Leave-one-out sensitivity analysis investigating the effect of ALT on diabetic nephropathy.


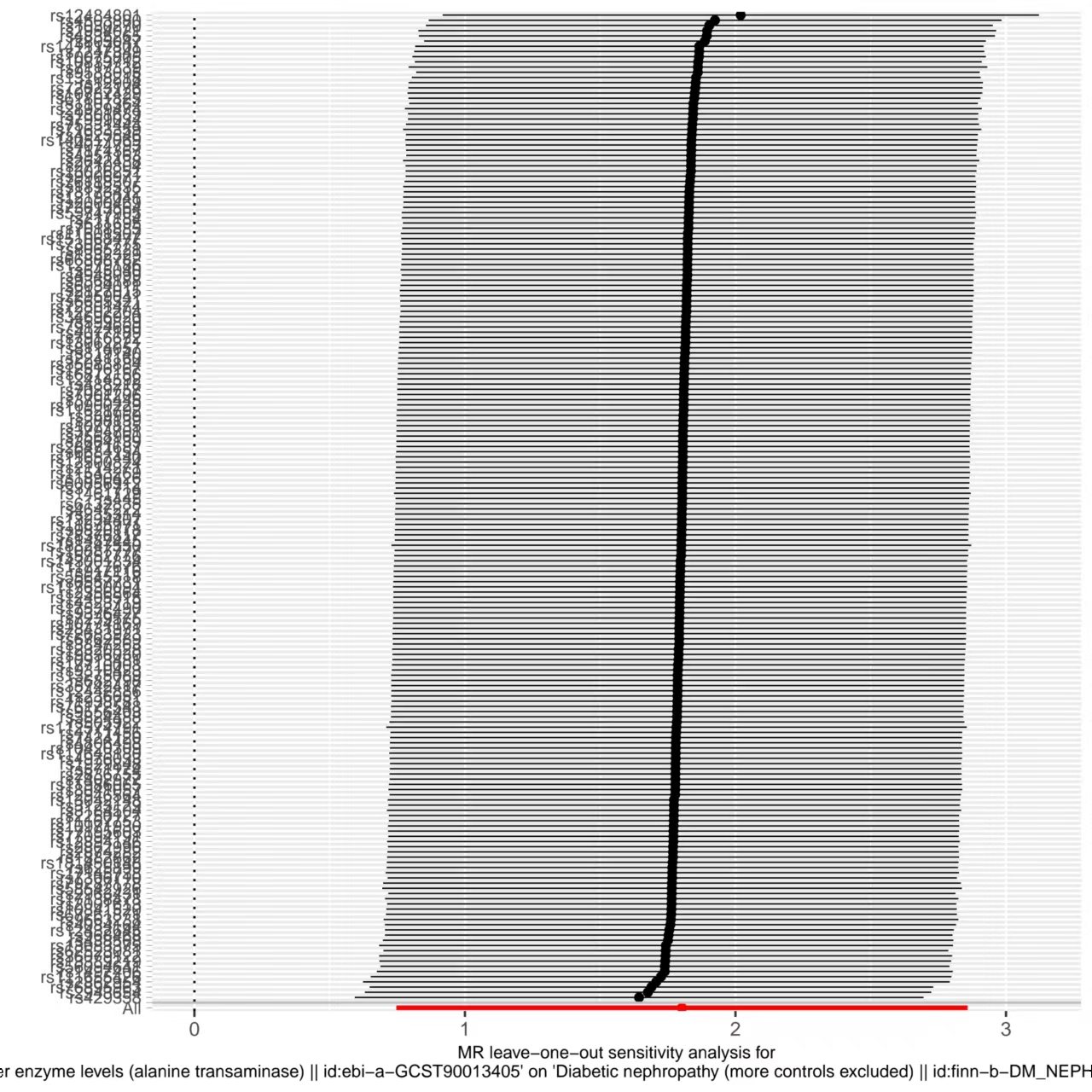


Figure S1D. Funnel plot of the main MR study investigating the effect of ALT on diabetic nephropathy.


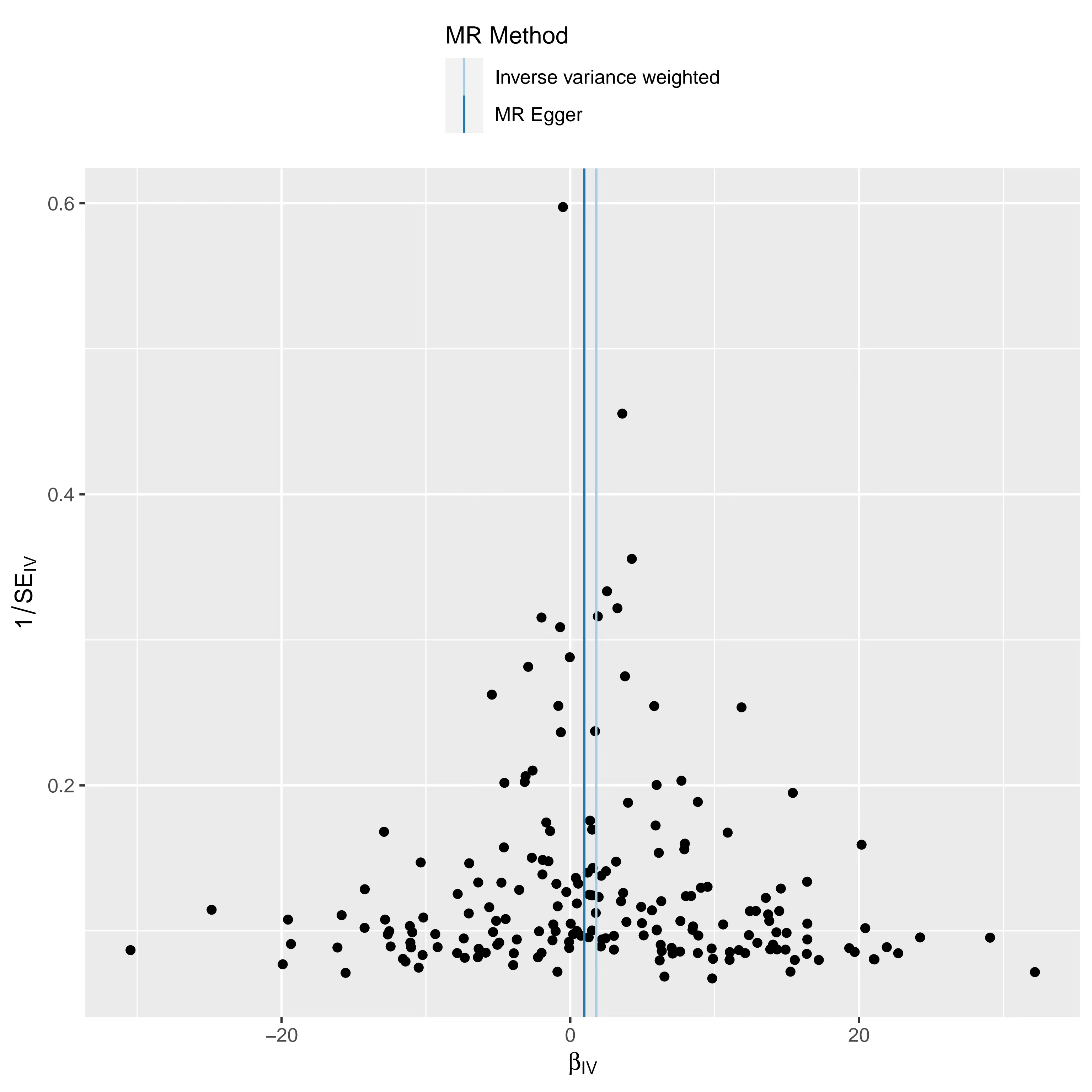

Supplement: Supplementary file 1 [file DataSheet_1.doc]
