# Supplementary material for: The association of alanine aminotransferase and diabetic microvascular complications: A Mendelian randomization study
Source: Front Endocrinol (Lausanne). 2023 Jan 19;14:1104963. doi: 10.3389/fendo.2023.1104963 (PMC9892708; doi:10.3389/fendo.2023.1104963)

Figure S2A. A two-sample MR study of the effect of ALT on diabetic retinopathy.


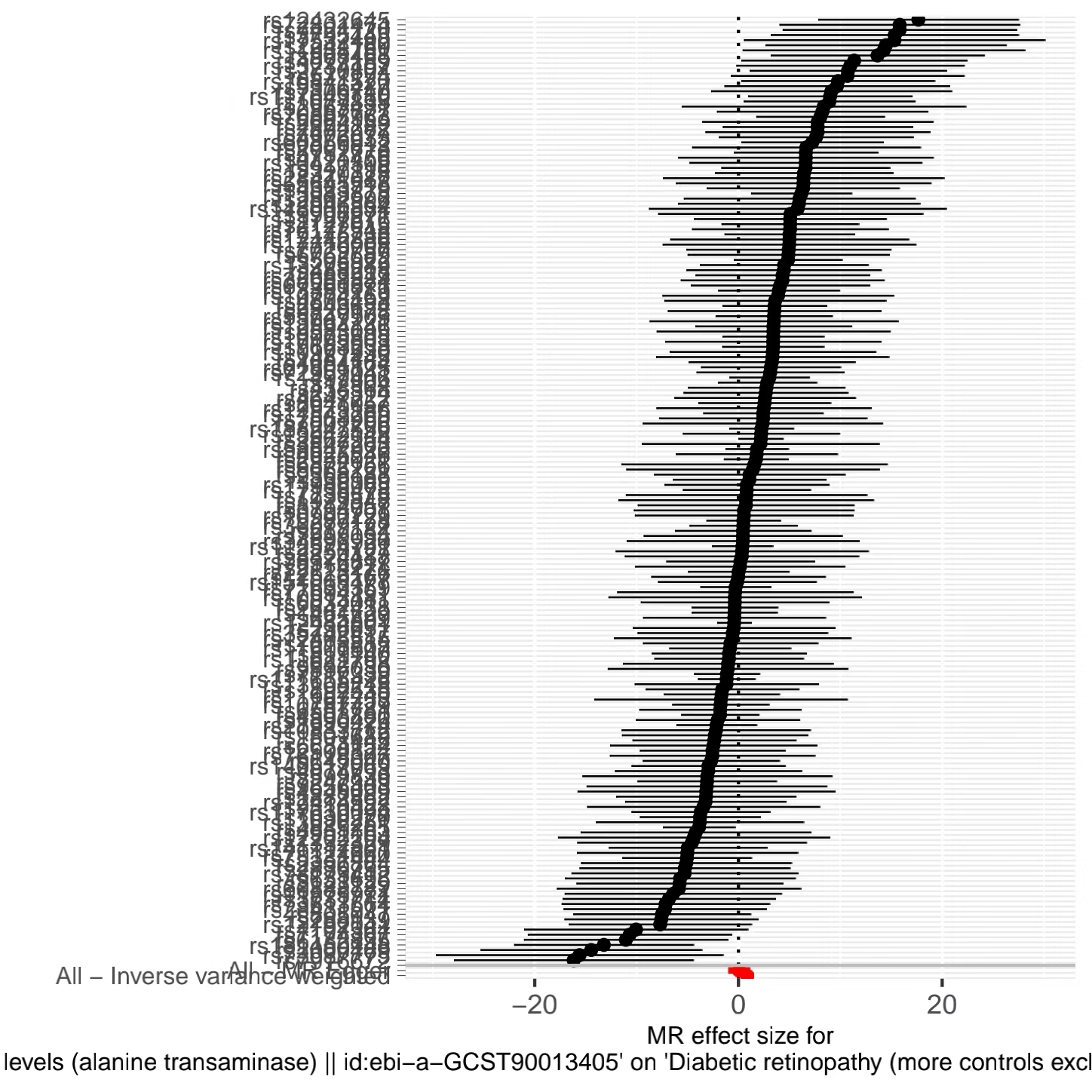


Figure S2B. Scatter plot of the main MR study investigating the effect of ALT on diabetic retinopathy.


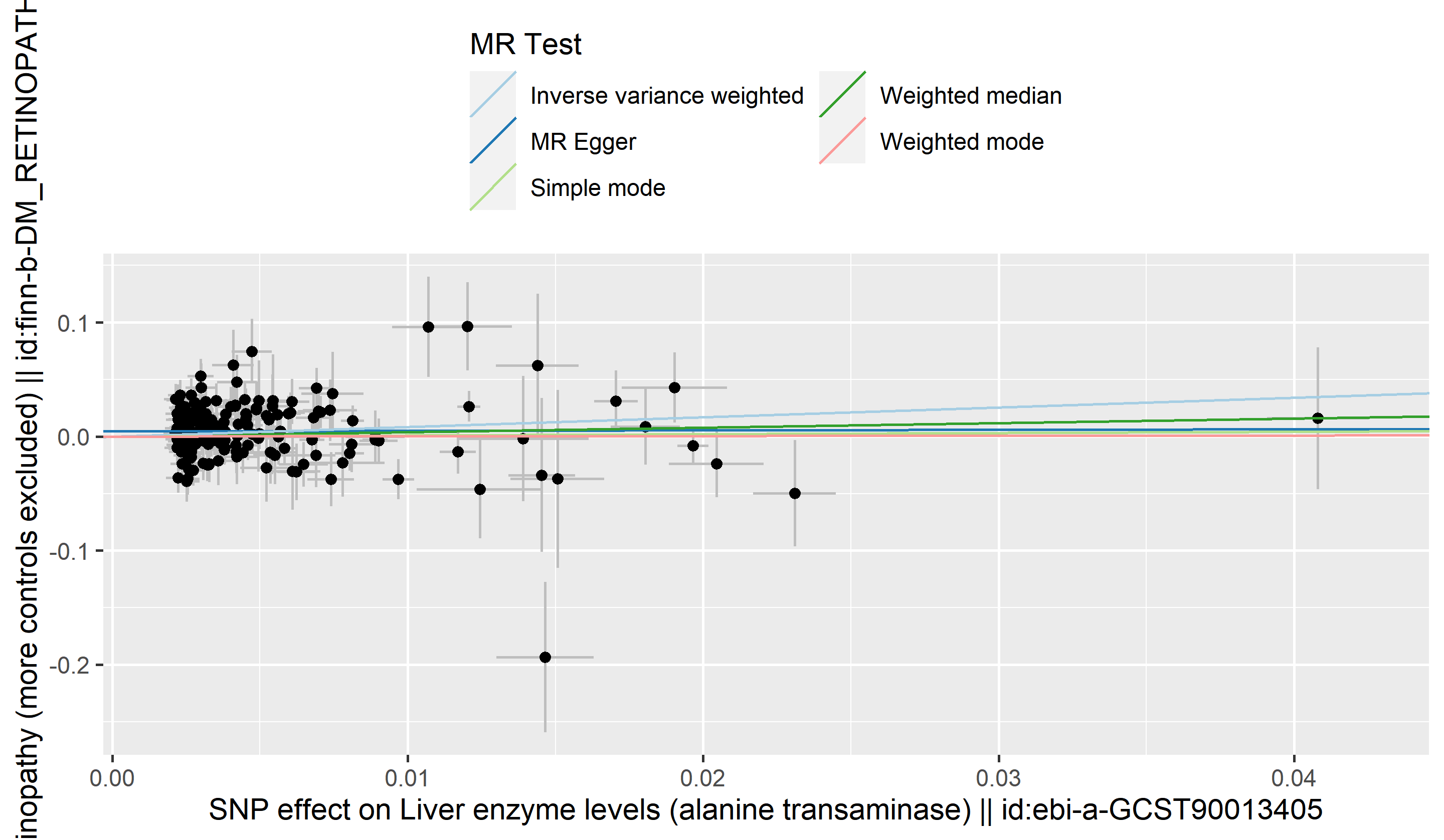


Figure S2C. Leave-one-out sensitivity analysis investigating the effect of ALT on diabetic retinopathy.


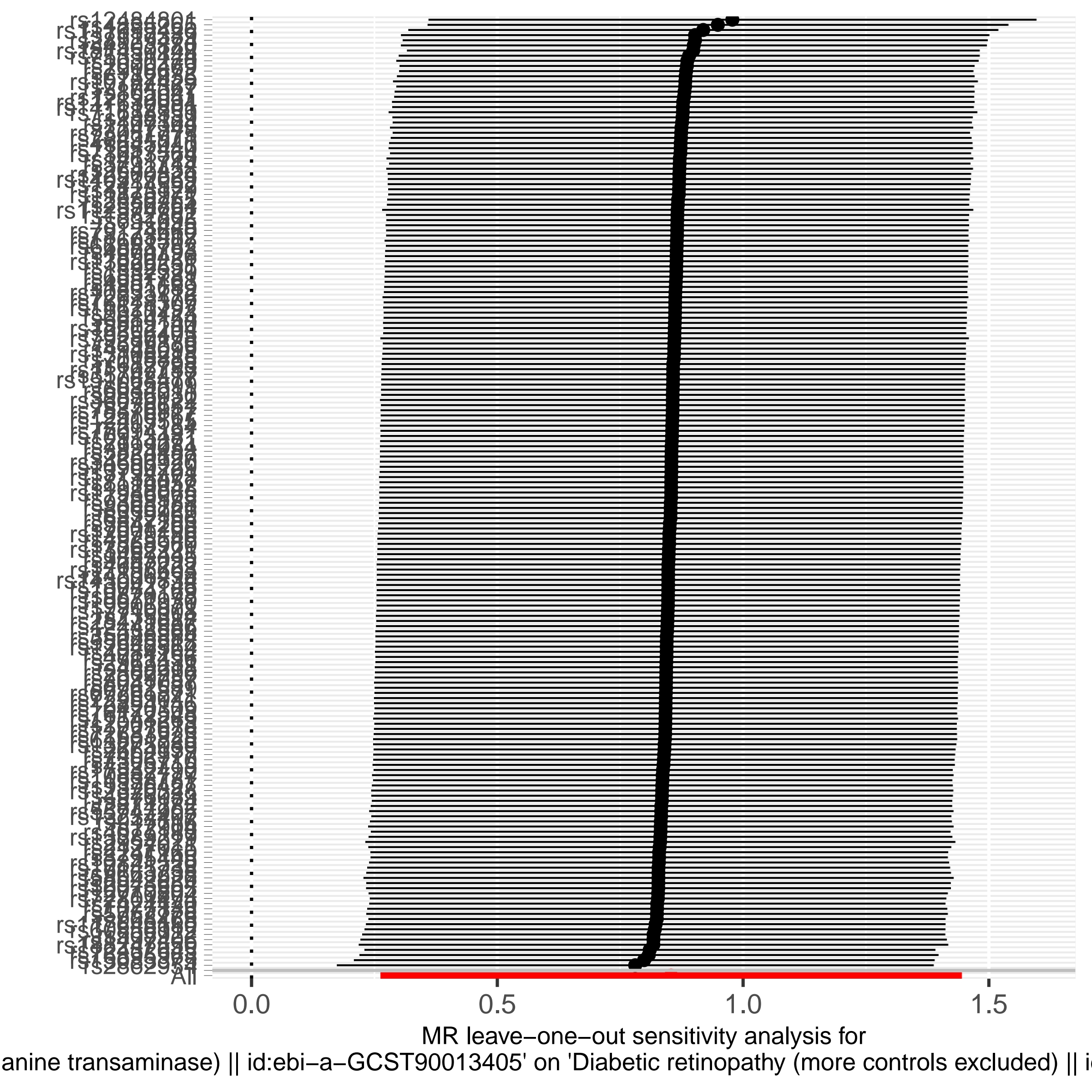


Figure S2D. Funnel plot of the main MR study investigating the effect of ALT on diabetic retinopathy.


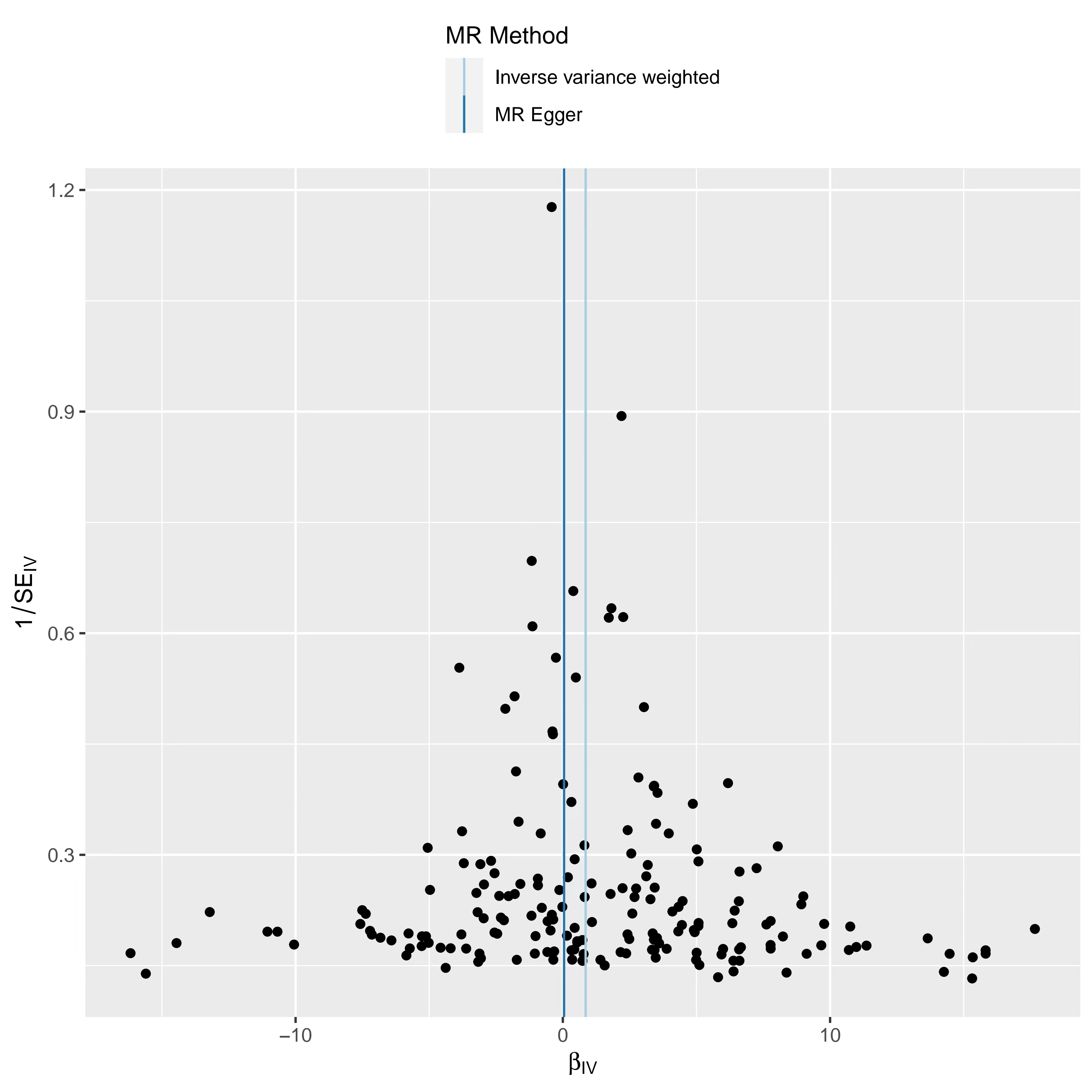

Supplement: Supplementary file 2 [file DataSheet_2.doc]
